# Identification and Validation of Mitophagy‐Related Biomarkers in Colorectal Cancer: An Integrated Analysis of Single‐Cell Transcriptome and Mendelian Randomization

**DOI:** 10.1155/genr/5579542

**Published:** 2026-05-11

**Authors:** Jingyi Zhao, Meng Kong, Sining Wang, Zhixin Cao, Xiangguo Tian

**Affiliations:** ^1^ Department of Pathology, Shandong Provincial Hospital Affiliated to Shandong First Medical University, Jinan, 250021, Shandong, China, sph.com.cn; ^2^ Department of Gastrointestinal Surgery, Shandong Provincial Hospital Affiliated to Shandong First Medical University, Jinan, 250021, Shandong, China, sph.com.cn; ^3^ Department of Gastroenterology, Shandong Provincial Hospital Affiliated to Shandong First Medical University, Jinan, 250021, Shandong, China, sph.com.cn

**Keywords:** biomarkers, colorectal cancer, mitophagy, single-cell RNA sequencing, two-sample Mendelian randomization

## Abstract

Mitophagy is essential for cancer formation and invasion, but its role in colorectal cancer (CRC) remains unclear. We obtained sequencing data and mitophagy‐related genes (MP‐RGs) from public databases. Differential expression analysis and weighted gene coexpression network analysis (WGCNA) identified mitophagy‐related differentially expressed genes (DE‐MPGs). Mendelian randomization (MR) analysis identified candidate genes with genetically supported causal relevance to CRC. Biomarkers were identified using machine learning, receiver operating characteristic (ROC) analysis and expression studies. Single‐cell RNA sequencing (scRNA‐seq) analyzed biomarker expression profiles in various CRC cell types. Quantitative PCR (qPCR) validated biomarker expression in clinical CRC samples. 147 DE‐MPGs were identified. MR analysis revealed seven genes with potential causal contributions to CRC susceptibility. Three genes, SGCE (IVW: OR = 1.00041, *p* = 0.011), ATP8B2 (IVW: OR = 0.99920, *p* = 0.042), and RANGAP1 (IVW: OR = 0.99861, *p* = 0.002), were selected as biomarkers. Immune microenvironment and checkpoint differences were observed between CRC and controls. Biomarker expression varied among cell types. qPCR showed decreased SGCE and ATP8B2 and increased RANGAP1 in CRC. SGCE, ATP8B2, and RANGAP1 can serve as mitophagy‐related biomarkers with genetically supported causal relevance to CRC, providing new insights for CRC diagnosis and therapy.

## 1. Introduction

Colorectal cancer (CRC) is one of the most common malignant tumors globally, with rising morbidity and mortality rates, seriously threatening human health in recent years [1]. Although extensive CRC screening programs have detected an increasing number of early CRC cases, a lot of patients are still diagnosed at advanced stages, with 20% of them presenting with metastatic CRC [2]. The development of therapeutic regimens for metastatic CRC, such as cytotoxic chemotherapy, biologic therapies, immunotherapies (e.g., immune checkpoint inhibitors), and surgical procedures (e.g., robot‐assisted colorectal surgery) [3, 4], has yielded significant efficacy in a subset of CRC patients. As well as the theory proposing that the endosome–lysosome system is directly involved in the fundamental biological processes of CRC pathogenesis [5]. However, these therapies remain ineffective in some patients [6]. The prognosis for metastatic CRC remains very poor [2]. The molecular mechanisms of CRC invasion and metastasis remain incompletely understood. Therefore, exploring effective molecular biomarkers and novel potential therapeutic targets is crucial for early detection, personalized treatment, and improving the long‐term prognosis of CRC patients.

Mitophagy is a selective autophagy of damaged or excessive mitochondria, playing a vital role in maintaining cellular homeostasis and tumorigenesis [7]. Mitophagy is always induced by mitochondrial depolarization, hypoxia, and metabolic stress [8, 9]. It has been shown that mitophagy affects the metabolic reprogramming of mitochondria and the accumulation/clearance of damaged mitochondria in tumors through different mechanisms [10]. In tumors, mitophagy appears to have both pro‐ and anticancer functions, depending on the tumor type, stage, or its metabolic activity [9]. Previous studies have confirmed that defective mitophagy is implicated in the tumor progression of CRC. Several mitophagy‐correlated factors or drugs could modulate mitophagy, thereby promoting or inhibiting CRC progression [11–13]. In addition, relevant studies have also demonstrated that under endoplasmic reticulum stress, upregulation of LMTK2 enhances autophagy to maintain endoplasmic reticulum homeostasis, ultimately promoting the survival of CRC cells [14]. However, the function of mitophagy in CRC remains unclear. Identifying mitophagy biomarkers is helpful in designing mitophagy modulators for tumor therapy; thus, interventions targeting mitophagy may possess therapeutic potential in CRC.

RNA sequencing (RNA‐seq) enables comprehensive analysis of the transcription levels of all genes within cells or tissues and reveals the molecular mechanisms underlying the occurrence and development of diseases [15]. However, traditional RNA‐seq analysis can only reflect the correlation between genes and diseases, cannot exclude the interference of confounding factors and reverse causality bias, and is difficult to clarify the potential causal contribution of genes to diseases. Mendelian randomization (MR) uses single‐nucleotide polymorphisms (SNPs) as instrumental variables and simulates the principle of randomized controlled trials, which can effectively infer the causal association between modifiable exposure factors and outcome indicators and simultaneously evaluate the independent and combined effects of multiple exposure factors. Notably, it cannot directly demonstrate causality at the molecular or cellular mechanistic level or fully encompass complex biological interactions [16, 17]. Combining MR with RNA‐seq can exactly compensate for the shortcomings of “correlation analysis” and verify the causal relationship between mitophagy‐related genes (MP‐RGs) and CRC at the population genetics level. Nevertheless, the combined analysis of the two lacks cell‐specific resolution of gene expression, and the occurrence and progression of CRC rely on heterogeneous interactions among multiple cells in the tumor microenvironment (TME) [18]. Expression analysis at the single‐tissue level fails to reveal gene functions in specific cell types. Single‐cell RNA sequencing (scRNA‐seq) can accurately quantify gene expression levels in individual cells, facilitate the analysis of cellular heterogeneity, and reveal gene expression differences between different cell types or within the same cell type under different conditions, which can precisely fill this gap [19]. Therefore, the integration of the three approaches in CRC research allows a comprehensive exploration from gene expression patterns to causal inference and then to cellular heterogeneity. It can more specifically screen mitophagy‐related biomarkers for CRC and provide more reliable molecular evidence for the early diagnosis and targeted therapy of CRC.

In conclusion, in this study, we, for the first time, integrated scRNA‐seq with MR analysis to establish a comprehensive research framework from gene screening to causal validation. Through single‐cell technology, we analyzed the cell‐specific expression patterns of MP‐RGs within the microenvironment of CRC. Using the MR analysis, we were the first to validate the causal associations between three genes (SGCE, ATP8B2, and RANGAP1) and CRC, breaking through the limitations of traditional correlation studies. The research paradigm of “omics screening ⟶ causal validation ⟶ single‐cell localization ⟶ clinical validation” provides new ideas for the discovery of tumor markers.

## 2. Results

### 2.1. Recognition of 147 DE‐MPGs in CRC

In TCGA‐COAD, 5423 differentially expressed genes (DEGs) in CRC were identified, including 2866 upregulated and 2557 downregulated genes (Figures 1(a) and 1(b)). Remarkably, the GSVA score in CRC was higher than that in controls (*p* < 0.05) (Figure 1(c)). Clustering analysis revealed that there were no outlier samples (Supporting Figure S1). Afterward, all genes were divided into six modules (*β* = 18) (Figures 1(d) and 1(e)). It was noted that blue (cor = 0.42) and yellow (cor = −0.61) modules exhibited the most positive and negative correlations with the GSVA scores, respectively (*p* < 0.05) (Figure 1(f)). From these two modules, 517 key module genes were selected (|MM| > 0.8, |GS| > 0.2) (Figure 1(g)). At last, 147 DE‐MPGs were acquired by overlapping 5423 DEGs and 517 key module genes (Figure 1(h)).

FIGURE 1Recognition of DE‐MPGs in CRC. (a) Volcano map of differential gene. The x‐coordinate represented the difference multiple, and the y‐coordinate represented −log10(p). The larger the value was, the higher the significance. The red dots indicate upregulated genes, and the cyan dots indicate downregulated genes. (b) Heatmap of differential gene. The next section presents a heatmap of the expression levels of the top 10 upregulated and downregulated genes in the samples. The horizontal axis represents the samples, while the vertical axis represents the genes. The blue areas above indicate the normal samples, and the red areas indicate the CRC patient samples. Red denotes high‐expression genes, and blue denotes low‐expression genes. The previous section showed a density heatmap of the expression levels of the top 10 upregulated and downregulated genes in the samples. (c) Cloud and rain map of MP‐RGs. (d) Screening diagram of soft threshold. The horizontal axis represents the value of the weight parameter power, and the vertical axis of the left figure has no scale R2. The higher the square of the correlation coefficient, the closer the network was to the scale‐free distribution. The vertical axis of the right figure represents the average degree of all genes in the corresponding gene module. (e) Cluster dendrogram. The upper part shows the hierarchical clustering tree diagram of genes, and the lower part displays the gene module. (f) Heatmap of module–disease correlation. (g) Left: The scatter plot displays the GS and MM in the blue module. Right: The scatter plot shows the GS and MM in the yellow module. The x‐coordinate represents the correlation between genes and modules (MM), and the y‐coordinate indicates the correlation between genes and traits (GS). (h) Venn diagram of MP‐RDEGs.

**Figure:**
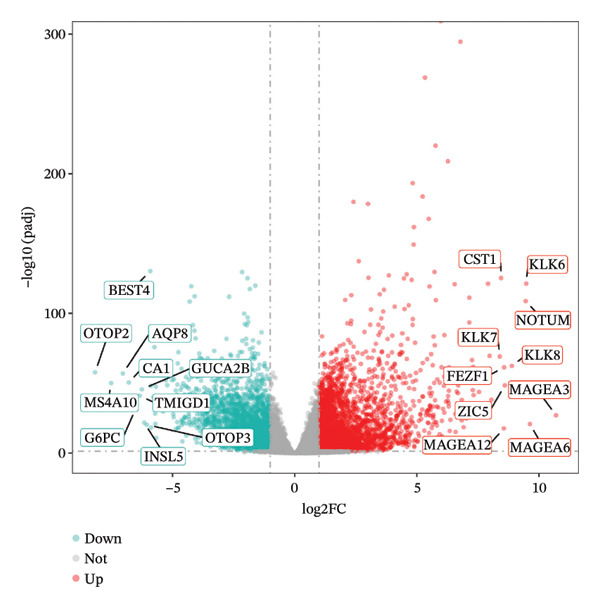
(a)

**Figure:**
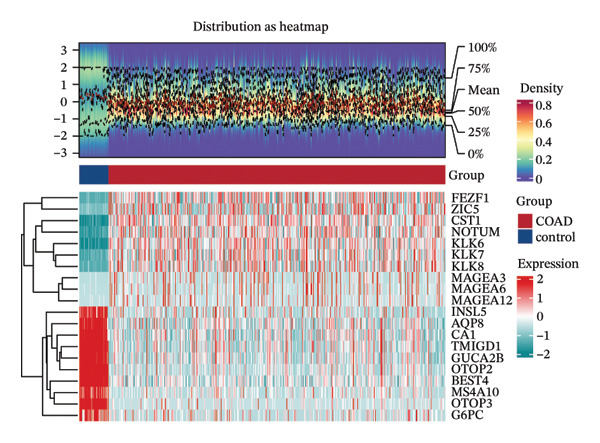
(b)

**Figure:**
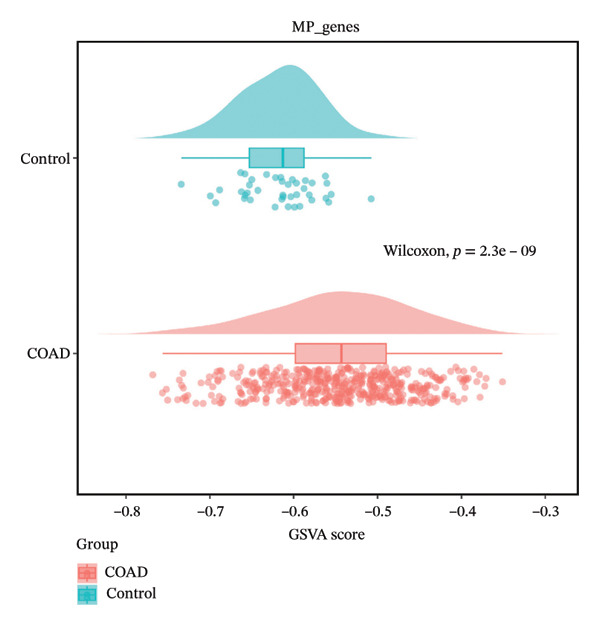
(c)

**Figure:**
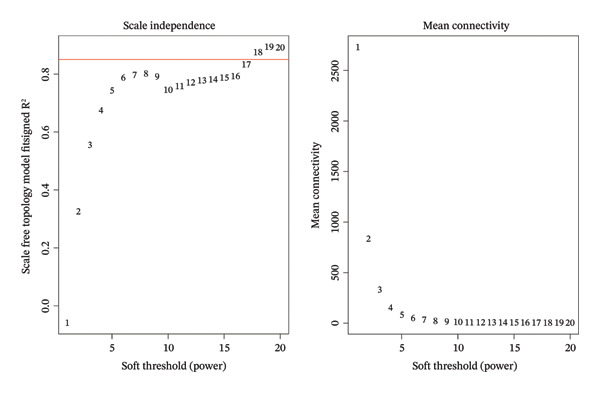
(d)

**Figure:**
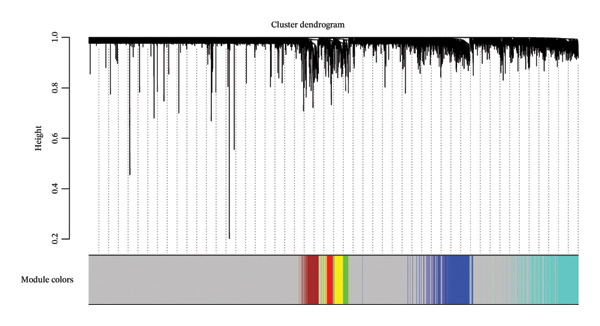
(e)

**Figure:**
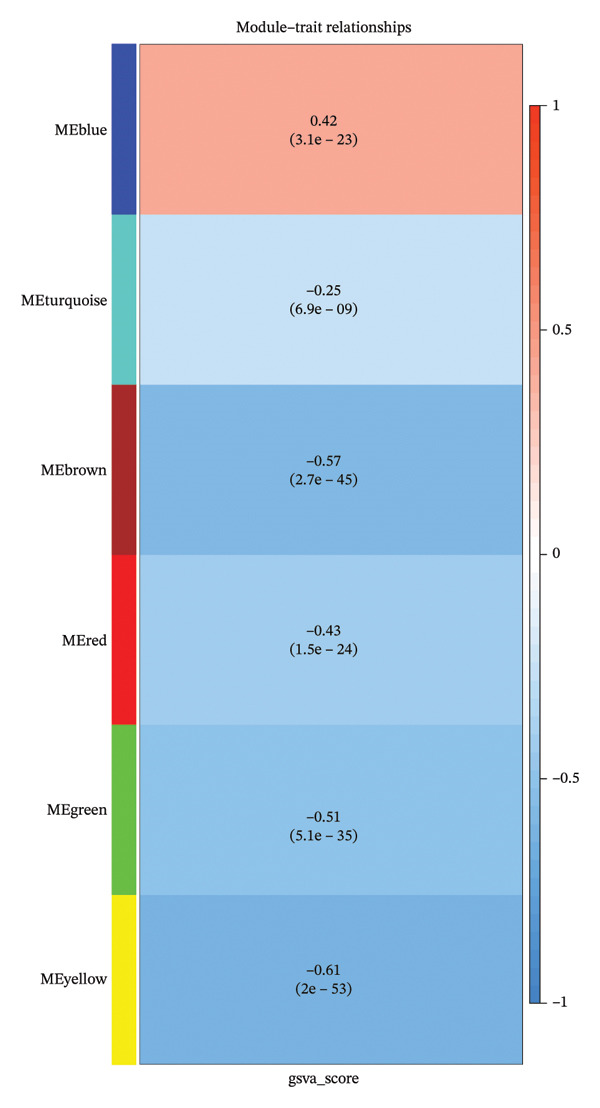
(f)

**Figure:**
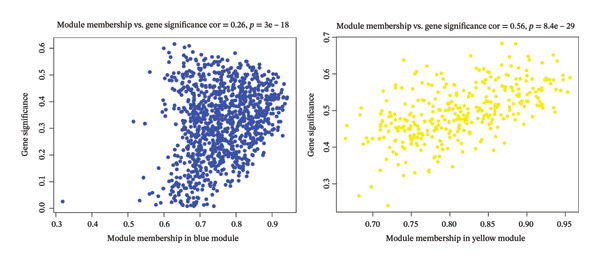
(g)

**Figure:**
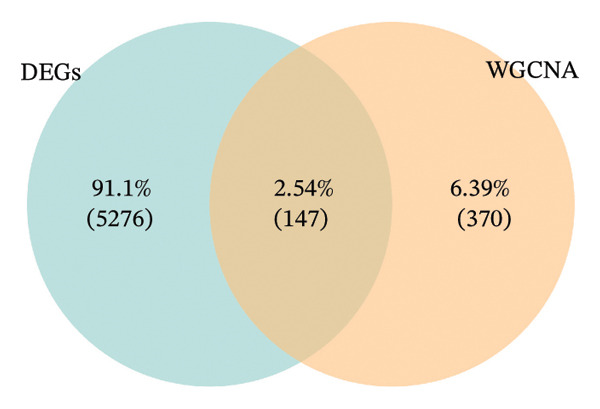
(h)

### 2.2. Acquisition of Seven Candidate Genes With Genetically Supported Causal Relevance to CRC

In two‐sample MR, 147 DE‐MPGs were exposure factors, and the CRC was an outcome factor. A total of 346 instrument variable (IVs) corresponding to 50 exposure factors (genes) were screened for the subsequent analysis (Supporting Table S1). MR Steiger filtering results showed that these 346 IVs would only affect CRC in one direction (Supporting Table S2). There were seven exposure factors (genes) with genetically supported causal relevance to CRC (PIVW < 0.05) (Table 1). Among them, SGCE and TSPYL5 were risk factors (OR > 1), while RANGAP1, SYT11, ATP8B2, STK32B, and IFRD2 served as protective factors (OR < 1) (Figure 2(a)). Slopes (IVW test) of exposure factors (genes) with protective factors were positive in scatter plots. Otherwise, the risk factors were negative (Figure 2(b)). Meanwhile, the forest plots showed that five exposure factors’ IVW loci were to the right of the 0, indicating that they also were risk factors; conversely, two exposure factors’ IVW loci to the left of the 0 were protective factors (Supporting Figure S2). Additionally, the IVs of seven exposure factors (genes) were evenly distributed on both sides of the solid line, which showed that the result satisfied Mendel’s second law of random grouping (Supporting Figure S3). Fortunately, there was no heterogeneity and horizontal pleiotropy in the seven exposure factors (genes) (*p* > 0.05) (Supporting Table S3, S4). LOO analysis again proved the stability of the above results (Supporting Figure S4). To sum up, the seven exposure factors (genes) were considered candidate genes in CRC for further analysis. Notably, candidate genes were linked with GO terms such as the trans‐Golgi network, early phagosome, and SUMO ligase complex (Figure 2(c), Supporting Table S5). These results aided in understanding the multiple roles of mitophagy in CRC progression. What is more, a gene–gene interaction GGI network was built based on the seven candidate genes, illustrating a total of 66 gene–gene pairs, including PEG10‐KL, TSPYL5‐ZNF518B, SGCE‐PPP1R9A, etc. (Figure 2(d), Supporting Table S6). This network uncovered the manifold interactions of candidate genes and suggested that biomarkers might influence CRC progression by affecting manifold GGI.

**TABLE 1:** The analysis result of MR evaluation.

Outcome	Exposure	Method	nsnp	b	se	pval	lo_ci	up_ci	or	or_lci95	or_uci95	Symbol
Colon cancer || id:ukb‐b‐20145	|| id:eqtl‐a‐ENSG00000100401	Inverse variance weighted	7	−0.001394284	0.000445827	0.001763515	−0.002268104	−0.000520464	0.998606687	0.997734466	0.999479671	RANGAP1
Colon cancer || id:ukb‐b‐20145	|| id:eqtl‐a‐ENSG00000127990	Inverse variance weighted	10	0.000411317	0.000161034	0.010642671	9.57E‐05	0.000726945	1.000411402	1.000095694	1.000727209	SGCE
Colon cancer || id:ukb‐b‐20145	|| id:eqtl‐a‐ENSG00000132718	Inverse variance weighted	19	−0.00033689	0.000162176	0.037773013	−0.000654755	−1.90E − 05	0.999663167	0.999345459	0.999980975	SYT11
Colon cancer || id:ukb‐b‐20145	|| id:eqtl‐a‐ENSG00000143515	Inverse variance weighted	5	−0.000797001	0.000392293	0.042189225	−0.001565895	−2.81E − 05	0.999203316	0.99843533	0.999971893	ATP8B2
Colon cancer || id:ukb‐b‐20145	|| id:eqtl‐a‐ENSG00000152953	Inverse variance weighted	3	−0.00270895	0.001311164	0.038822344	−0.005278831	−0.000139069	0.997294716	0.994735078	0.999860941	STK32B
Colon cancer || id:ukb‐b‐20145	|| id:eqtl‐a‐ENSG00000180543	Inverse variance weighted	3	0.001512681	0.000715643	0.034537873	0.00011002	0.002915342	1.001513826	1.000110026	1.002919596	TSPYL5
Colon cancer || id:ukb‐b‐20145	|| id:eqtl‐a‐ENSG00000214706	Inverse variance weighted	4	−0.001339791	0.000606892	0.027270239	−0.002529299	−0.000150283	0.998661106	0.997473897	0.999849728	IFRD2

FIGURE 2MR analysis and enrichment analysis of candidate genes. (a) MR analysis result diagram of seven exposure factors genes. (b) Scatter plot of correlation analysis of seven exposure factors genes. (c) Bar chart of GO analysis results of candidate genes (blue represents the cellular component, and red represents the molecular function). (d) Diagram of candidate genes related to gene function and the functions involved. The large circle indicates the candidate genes, and the small circles represents genes related to the candidate genes.

**Figure:**
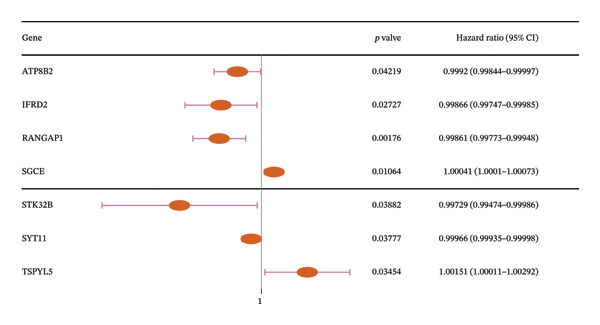
(a)

**Figure:**
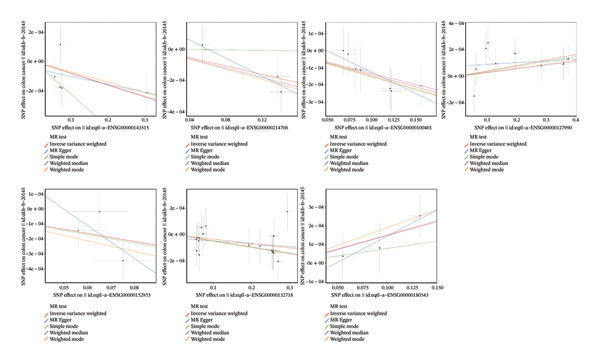
(b)

**Figure:**
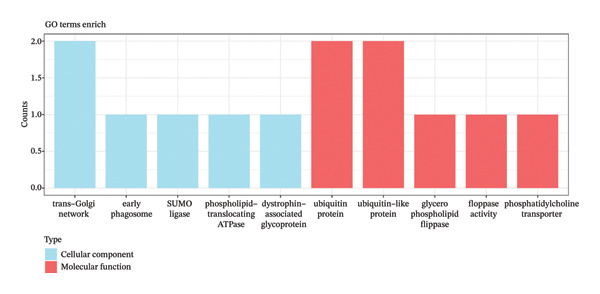
(c)

**Figure:**
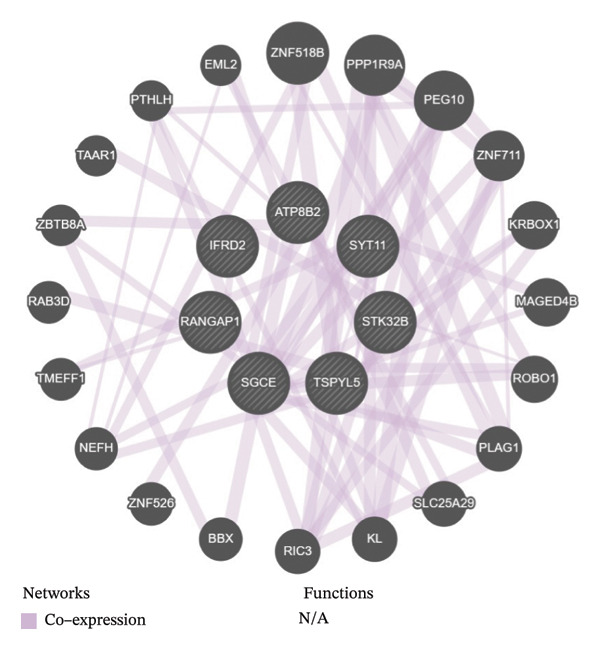
(d)

### 2.3. RANGAP1, SGCE, and ATP8B2 Were Effective Biomarkers for CRC

Based on Least Absolute Shrinkage and Selection Operator (LASSO) regression analysis, six feature genes were screened (lambda.min = 0.00315) (Figure 3(a)). In addition, five feature genes were identified through the SVM‐RFE algorithm (Figure 3(b)). Next, from the intersection of these feature genes, four hub genes were acquired, including RANGAP1, SGCE, ATP8B2, and STK32B (Figure 3(c)). Hub gene expression levels in training and testing datasets were illustrated (Supporting Figure S5). The expression profiles of all hub genes except STK32B had significant differences, and the trends were consistent. AUC values of all hub genes except STK32B exceeded 0.7 (Supporting Figure S6‐S7). In a word, RANGAP1, SGCE, and ATP8B2 were ultimately selected as mitophagy‐related biomarkers for CRC. Subsequently, a nomogram integrating biomarkers was developed (Figure 3(d)). In addition, ROC curves indicated that the nomogram model could well predict the occurrence of CRC (Figure 3(e)). In addition, DCA curves demonstrated that the net benefit of this nomogram model was notably higher, revealing outstanding clinical utility (Figure 3(f)). In summary, this nomogram model held potential as an effective tool for clinical decision‐making in managing CRC, with biomarkers demonstrating superior diagnostic value. Additionally, biomarker‐related functional pathways were examined by gene set enrichment analysis (GSEA) (Figure 3(g)). It was worth mentioning that biomarkers were coenriched in pathways such as the neuroactive ligand–receptor interaction pathway. In summary, mitophagy might exert a critical role in CRC progression by modulating these biomarker‐related pathways.

FIGURE 3Determination of biomarkers in CRC. (a) The LASSO regression analysis result graph. (The left figure demonstrates the tenfold cross‐validation of adjusting parameters in LASSO analysis. The *x* axis represents the logarithm of lambda, and the *y*‐axis represents the model error. The number of variables is indicated at the top of the *x*‐axis, with the number of independent variables decreasing as lambda increased. The right figure presents the LASSO coefficient spectrum. The *x*‐axis shows the logarithm of lambda, and the *y*‐axis shows the coefficients of the variables, with each line representing a gene.) (b) The SVM‐RFE analysis result diagram. The x‐coordinate indicates the number of characteristic genes, and the y‐coordinate reflects the prediction accuracy of the model. (c) The Venn diagram illustrates the intersection of the genes obtained by LASSO and SVM‐RFE analysis. (d) The nomogram of biomarkers is constructed. (e) The ROC curve graph of the nomogram of biomarkers is generated. The closer the AUC is to 1, the more accurate the diagnosis was. When 0.7 > AUC > 0.5, the diagnostic accuracy is relatively low; when 0.9 > AUC > 0.7, the diagnostic effect has some reference value; when AUC > 0.9, the diagnostic accuracy is high; and when AUC = 0.5, there is no diagnostic value. (f) The DCA curve graph of the nomogram model is plotted. (g) The gene set enrichment analysis diagram of RANGAP1, SGCE, and ATP8B2. Each polyline represents a pathway, with the peak of each polyline indicating the enrichment fraction of that pathway. The genes before the peak are considered core genes within the pathway gene set. A peak in the upper left corner indicates that the core genes are primarily upregulated based on the differential expression analysis of biomarkers. Conversely, a peak in the lower right corner suggests that the core genes are primarily downregulated based on the differential expression analysis of biomarkers.

**Figure:**
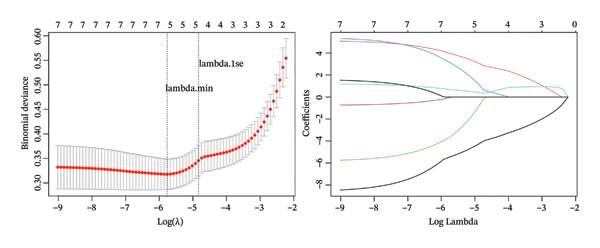
(a)

**Figure:**
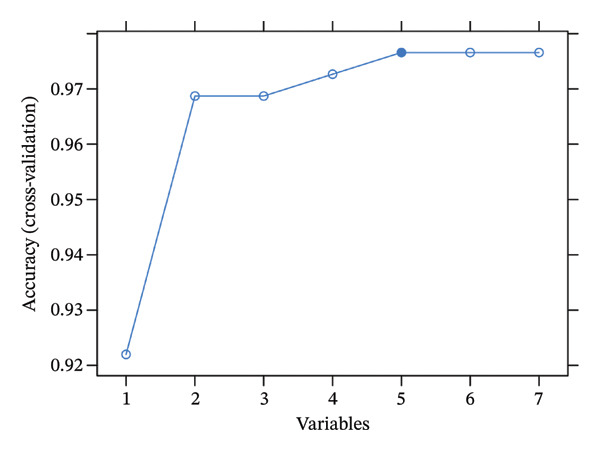
(b)

**Figure:**
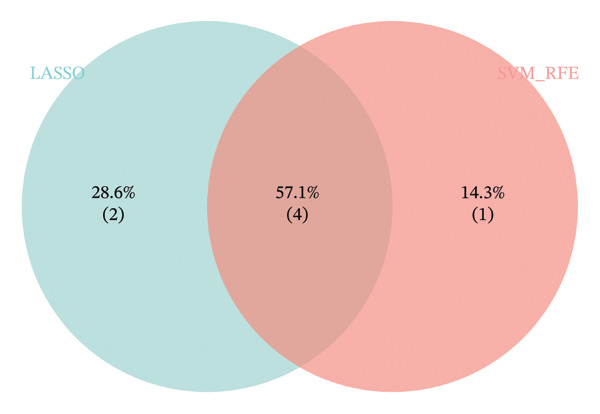
(c)

**Figure:**
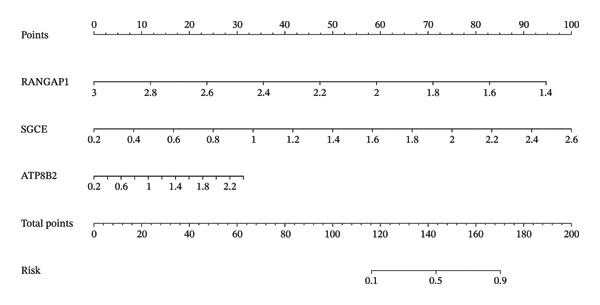
(d)

**Figure:**
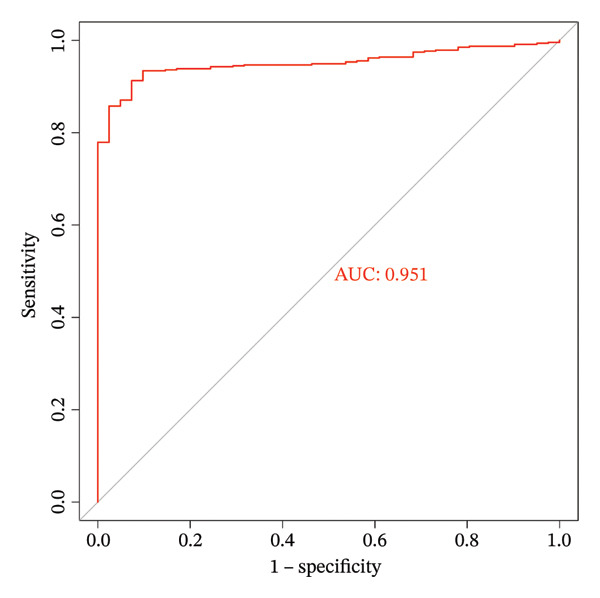
(e)

**Figure:**
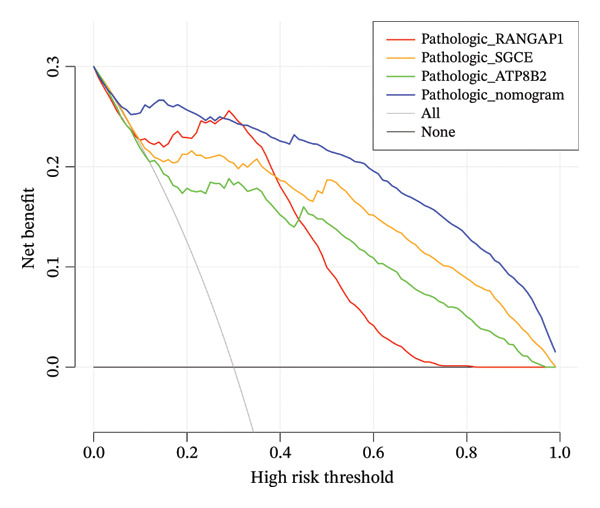
(f)

**Figure:**
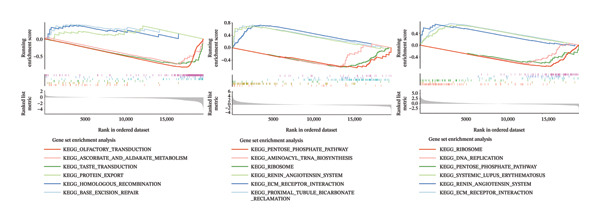
(g)

### 2.4. Different Immune Microenvironment in CRC and Potential Drugs Targeting Biomarkers

The infiltration profiles of 22 immune cell types are illustrated in Figure 4(a). There were 15 differential immune cells between the CRC and controls (*p* > 0.05) (Figure 4(b)). Correlation analyses showed that M2 macrophages (cor = 0.35) and activated memory CD4 T cells (cor = −0.31) demonstrated the strongest positive and inverse associations with SGCE, respectively (*p* < 0.05) (Figure 4(c)). What is more, there were 68 immune checkpoints, which showed significantly different expression between CRC and controls (*p* < 0.05) (Figure 4(d)). In particular, PVR showed the strongest positive correlation with RANGAP1, and CD160 demonstrated the strongest inverse correlation with ATP8B2 (cor = −0.34, *p* < 0.05) (Figure 4(e)). These biomarker‐mediated immune characteristics strikingly elucidated the association between the mitophagy and the immune microenvironment in CRC, offering valuable insights into the development of therapeutic strategies for CRC. The drug–gene network revealed 24 potential drugs targeting the biomarkers, such as butyl hydroxybenzoate‐RANGAP1, COUMESTROL‐ATP8B2, and cyclophosphamide‐SGCE (Figure 4(f)). These findings might provide clues for targeting mitophagy as a therapeutic approach for CRC.

FIGURE 4Immune microenvironment in CRC and potential drugs targeting biomarkers. (a) Map of immune cell infiltration abundance between disease and control. Each color represents a type of immune cell. (b) Diagram of the difference in infiltration levels between disease and control. Green represents the CRC group, and red represents the control group. (c) Left: Heatmap of correlation between biomarkers and differential immune cells. Middle: Scatter plot of negative correlation between biomarkers and immune cells. Right: Scatter plot of positive correlation between biomarkers and immune cells. (d) Diagram of differences between disease and control in immune checkpoint analysis. Blue represents the control group, and red represents the CRC group. (e) Left: Heatmap of correlation between biomarkers and differential immune checkpoint genes. Middle: Scatter plot of negative correlation between biomarkers and immune checkpoints. Right: Scatter plot of positive correlation between biomarkers and immune checkpoints. (f) Drug prediction map related to biomarkers. Orange represents the genes, and green represents the drugs.

**Figure:**
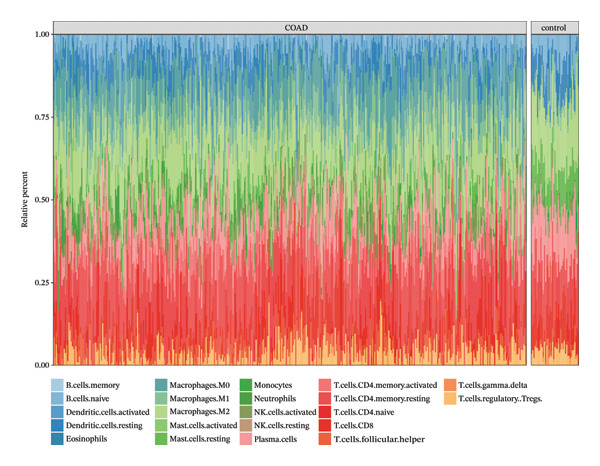
(a)

**Figure:**
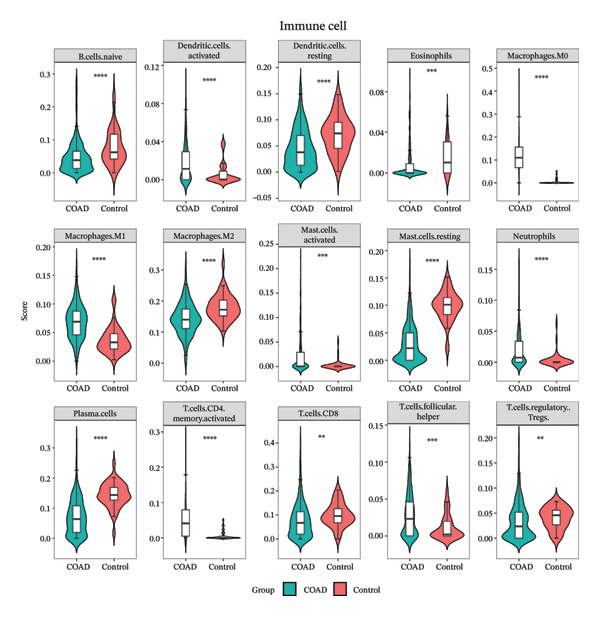
(b)

**Figure:**
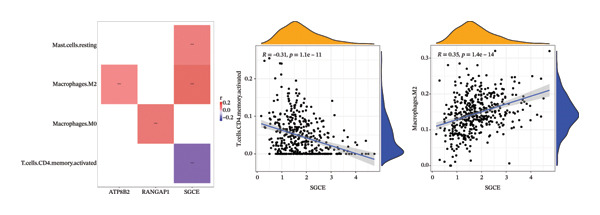
(c)

**Figure:**
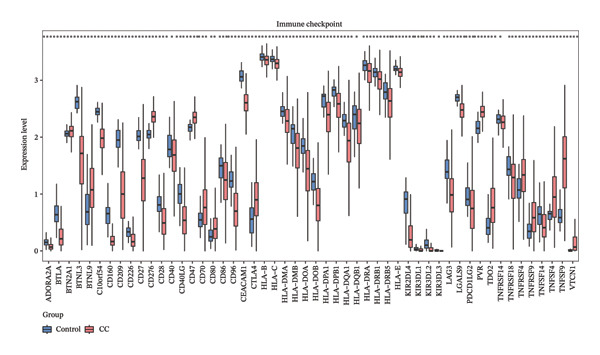
(d)

**Figure:**
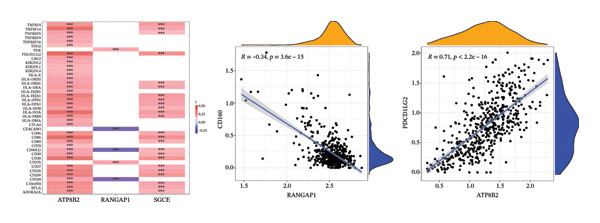
(e)

**Figure:**
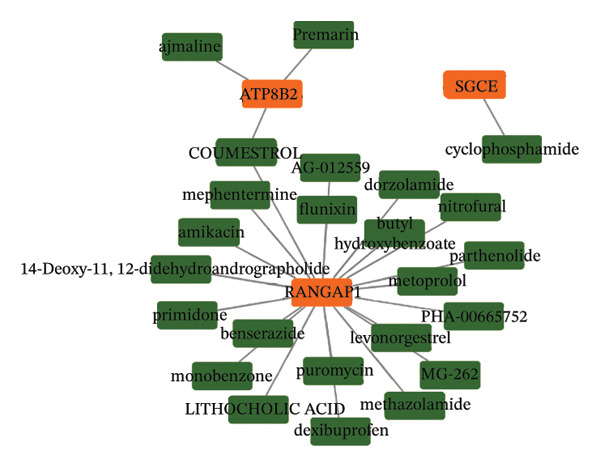
(f)

### 2.5. Altered Single‐Cell Level Expression of Biomarkers in CRC

After quality control, 28,009 high‐quality cells and 23,481 genes were retained after quality control (Figure 5(a)). According to the top 2000 HVGs and top 50 PCs, 19 cell clusters were generated (Figures 5(b), 5(c), 5(d), 5(e)). The average expressions of 19 cell cluster marker genes are shown in Supporting Figure S8. Afterward, eight distinct cell types were recognized, such as T cells, B cells, fibroblasts, and common myeloid progenitor (CMP) cells (Figure 5(f)). The marker genes of distinct cell types were specifically highly expressed in the corresponding cell types (Figure 5(g)). In particular, the CMP cells in CRC were significantly higher than in controls (Figure 5(h)). The delineation of cell‐type profiles offered important insights into the immune and cellular mechanisms involved in CRC. Between CRC and controls, the expression of RANGAP1 and SGCE in fibroblasts, the expression of SGCE and ATP8B2 in B cells, and the expression of ATP8B2 in T cells, all demonstrated marked differences (Supporting Figure S9‐S10). Differential biomarker expression might reflect functional specialization or dysfunction of distinct cell types in CRC.

FIGURE 5Single‐cell level expression of biomarkers. (a) The single‐cell data filtering diagram is displayed. (b) The scatter plot visualizes highly variable genes, with red representing high variable genes. (c) The principal component analysis diagram shows the two principal components on its axes, with blue indicating the disease group and red representing the control group. (d) Left: The PCA substitution test diagram is presented. Right: The scree plot of PCA analysis is shown, with the horizontal axis representing latitude values and the vertical axis showing standard deviation, while scatter points indicated dimension distribution. (e) The cell cluster diagram is generated, where different colors represent different cell clusters. (f) The cell annotation diagram is created, using different colors to distinguish various cell types. (g) The diagram illustrates specific gene expression in cells, where larger circles indicates higher expression levels. (h) The cluster cell difference diagram between CRC samples and control samples is displayed, with NS indicating no significance and ^∗^denoting *p* < 0.05.

**Figure:**
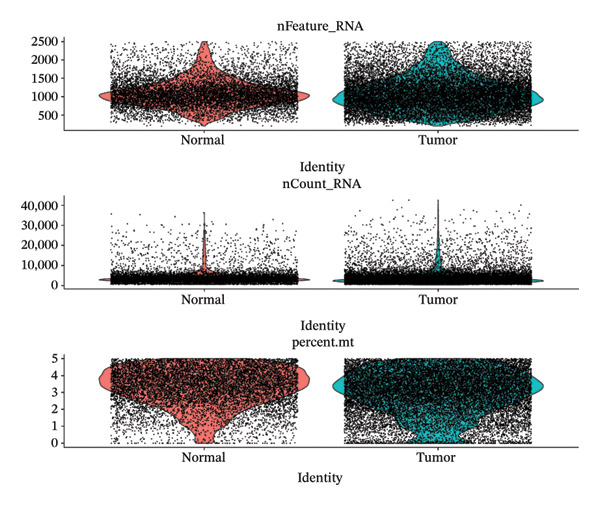
(a)

**Figure:**
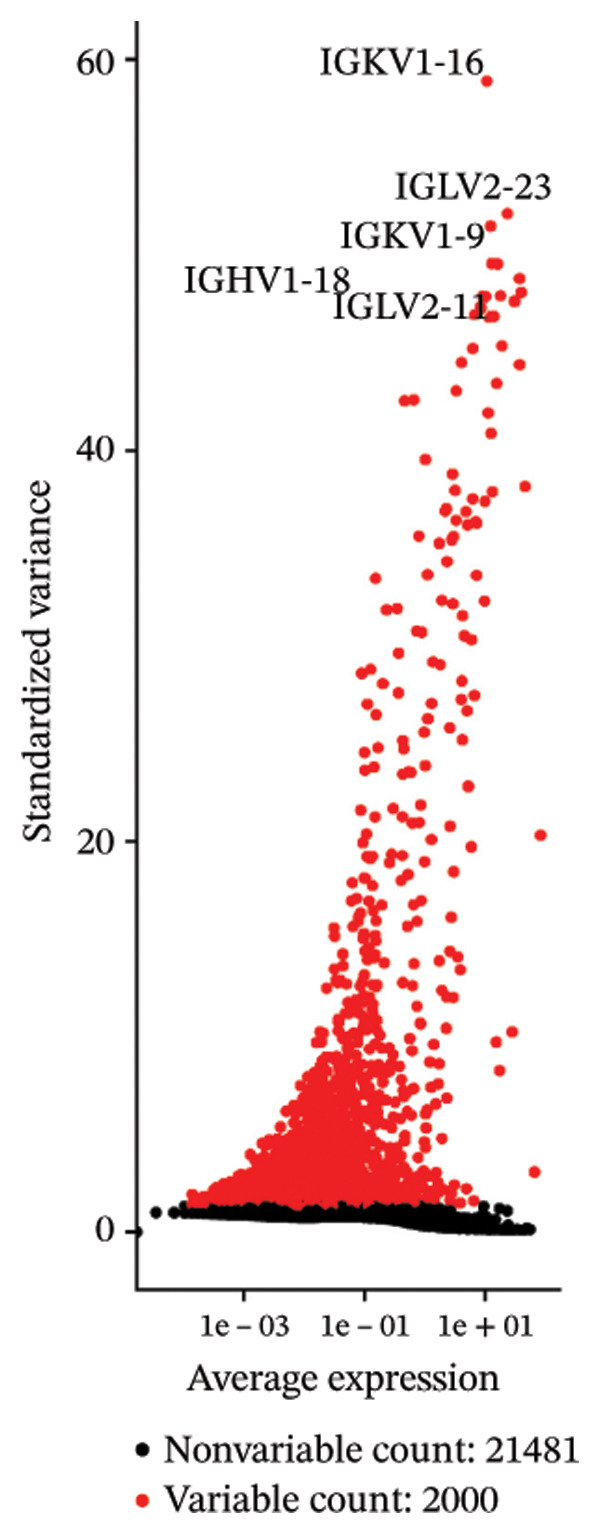
(b)

**Figure:**
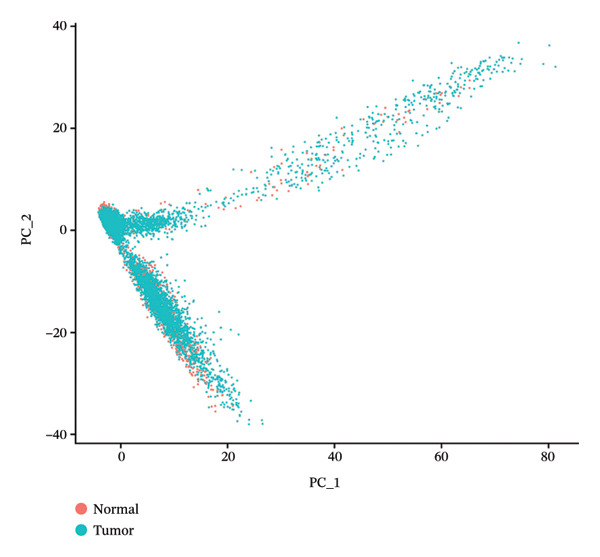
(c)

**Figure:**
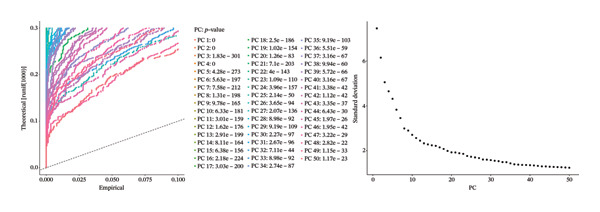
(d)

**Figure:**
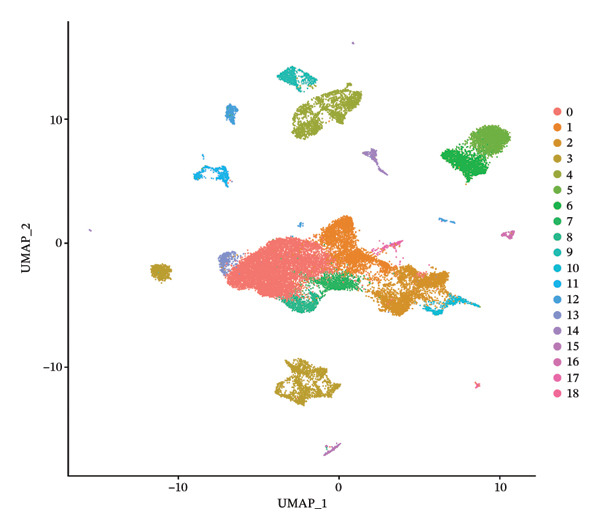
(e)

**Figure:**
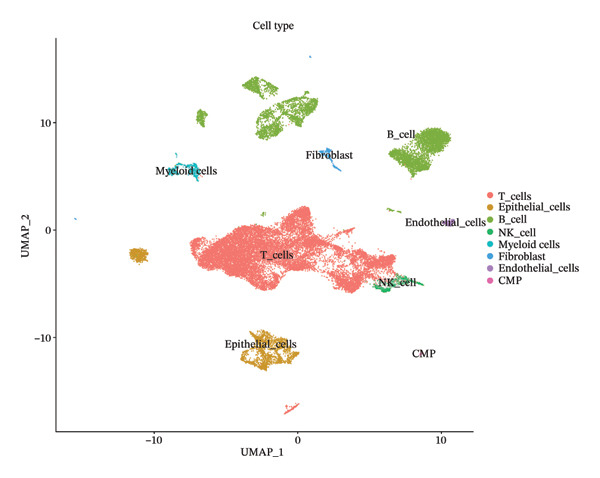
(f)

**Figure:**
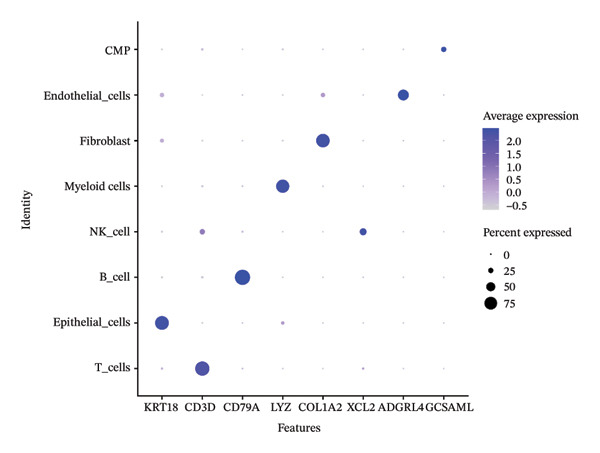
(g)

**Figure:**
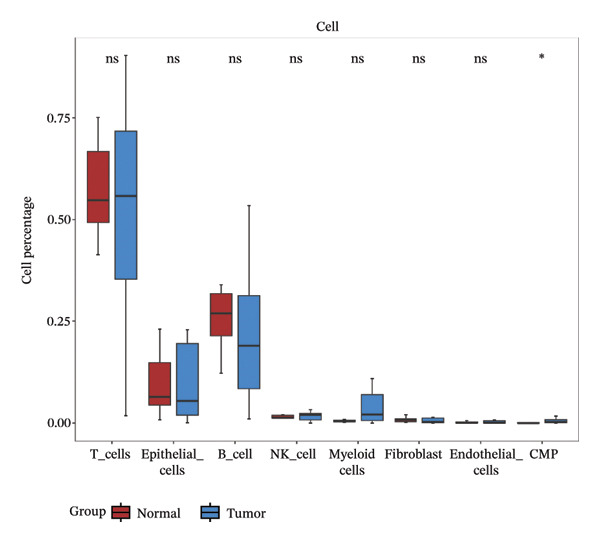
(h)

### 2.6. The Expression Level of Biomarkers in CRC Tissues by Quantitative PCR (qPCR)

**FIGURE 6:**
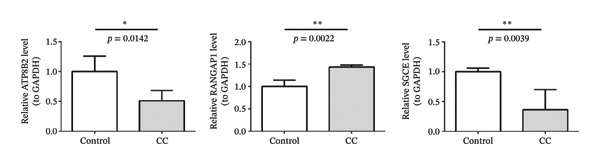
qPCR analysis of ATP8B2, RANGAP1, and SGCE expression levels in CRC tissues. ^∗∗^represents *p* < 0.01 and, ^∗^represents *p* < 0.05.

qPCR showed that ATP8B2 and SGCE exhibited decreased expression in CRC compared to the controls, while RANGAP1 expression in CRC was markedly higher (*p* < 0.05) (Figure 6). Differential expression of biomarkers further confirmed their clinical diagnostic value in CRC.

## 3. Discussion

Mitophagy is an important mitochondrial quality control process that eliminates damaged or excess mitochondria through autophagic degradation [7, 20]. Dysfunctional and excessive mitophagy has been linked to various diseases, including neurodegenerative disorders and cancer [20]. Several MP‐RGs have been implicated in tumor progression in CRC [7, 20]. The role of mitophagy in tumors is complex and depends on the type and stage of the tumor. In most of the studies, mitophagy‐related factors promote tumor progression by inhibiting mitophagy, revealing the tumor‐suppressive effects of mitophagy in CRC [11, 13]. Additionally, some drugs, including Tanshinone IIA (Tan IIA) and Oxymatrine, have suppressive effects on CRC through mitophagy induction in vitro and in vivo [21, 22]. On the other hand, mitophagy can be activated by low nutrient conditions during the progression of tumor to maintain mitochondrial metabolism for cell proliferation and play a critical role in colon cancer cell growth [23]. Thus, identifying the specific roles of the different mitophagy pathways underlying the occurrence and invasion of CRC is still a major challenge. The identification of mitophagy biomarkers will be beneficial for designing mitophagy modulators for tumors with high requirement for mitophagy. In this study, we identified three MP‐RGs (SGCE, ATP8B2, and RANGAP1) as potential biomarkers with genetically supported causal relevance to CRC susceptibility.

SGCE encodes the epsilon‐sarcoglycan protein, a member of the sarcoglycan family [24]. As a transmembrane protein, sarcoglycans link the muscle cytoskeleton to the extracellular matrix (ECM) [24]. SGCE is widely expressed in several different tissue types and regulates the accumulation and remodeling of the ECM [25]. SGCE has been reported to correlate with various cancers, including gastric and CRC [26, 27]. However, how it participates in the cancer progression remains unclear. In gastric cancer, SGCE expression was significantly lower in tumor tissues than in intestinal metaplasia tissues, suggesting its involvement in the transition from non‐neoplastic epithelium to gastric cancer [26]. Hypermethylated SGCE with decreased expression was associated with favorable survival in gastric cancer [26]. In CRC, SGCE expression was reduced in both MSI‐H tumors and MSI‐L/MSS tumors compared to nontumoral samples [27]. In our study, we also detected a lower expression of SGCE in CRC tissues versus the control group, aligning with prior reports [26, 27]. These results imply that reduced expression of SGCE may be associated with the progression of CRC. However, the role of SGCE still needs to be studied in depth.

The ATP8B2 is a type of Type‐IV P‐type ATPase (P4‐ATPase) that translocates specific lipids across the bilayer using the energy of ATP [28]. P4‐ATPases maintain membrane lipid asymmetry and regulate membrane trafficking, cytoskeletal dynamics, cell division, lipid metabolism, and lipid signaling [28]. ATP8B2 is implicated in a variety of pathophysiological conditions and has been described to be widely expressed in acetylated phospholipid‐rich tissues [29]. However, the literature on the role of ATP8B2 in human tumors is relatively scarce. A study has demonstrated that ATP8B2 is associated with poor prognosis in pancreatic cancer [30]. Additionally, research indicates that ATP8B2 expression is downregulated in bladder cancer and may serve as a prognostic biomarker for this disease [31]. Although there is no study directly indicating the expression and role of ATP8B2 in CRC, in this study, MR showed that ATP8B2 was a protective factor of CRC, and RT‐PCR showed that the expression level of ATP8B2 in CRC tissue was significantly lower than that in normal tissue. It could be inferred that the decrease in ATP8B2 expression may affect the membrane lipid regulation, thereby promoting tumor development. However, the role and potential mechanism of ATP8B2 in CRC still need further research.

RanGAP1 is considered to be a key protein in the transportation of RNA from the nucleus to the cytoplasm [32]. Regulation of RanGAP1 expression could affect RNA transportation, thereby regulating tumor cell function [33]. A recent study showed that RanGAP1 loss induces chromosomal instability and promotes the development of osteosarcoma [34]. On the other hand, in glioma cells, RanGAP1 downregulation causes nuclear entrapment of RanGTP, leading to intranuclear RNA accumulation and eventual tumor cell apoptosis [35]. In CRC, RanGAP1 is significantly overexpressed and correlates with poor patient prognosis. Further studies showed that METTL3/YTHDF1‐mediated N6‐methyladenosine modification of RanGAP1 promotes CRC progression via the MAPK pathway [36]. Our study also confirmed the high expression of RanGAP1 in CRC, which is consistent with the previous study. RanGap1 might become a diagnostic molecule and a potential therapeutic target of CRC.

Remodeling of the TME is a key driver of CRC progression, in which immune cell infiltration, immune checkpoint molecule expression, and activation of cancer‐associated fibroblasts (CAFs) collectively form a complex network that promotes tumor initiation and development [37, 38]. Regarding immune cell infiltration, this study identified 15 types of differentially infiltrated immune cells between CRC tissues and normal tissues, including increased infiltration of M2 macrophages and decreased infiltration of activated memory CD4+ T cells, which is consistent with the typical characteristics of the immunosuppressive microenvironment in CRC [39, 40]. Previous studies have shown that disordered mitophagy in CRC leads to massive accumulation of reactive oxygen species (ROS) and excessive secretion of immunoregulatory factors, indirectly exacerbating M2 macrophage infiltration [41, 42]. Activated memory CD4+ T cells are core antitumor adaptive immune cells in the CRC microenvironment. Under normal physiological conditions, they can be rapidly activated upon encountering tumor antigens and exert antitumor effects [43]. However, abnormal mitophagy results in insufficient energy metabolism in T cells, reduced secretion of antitumor factors, functional exhaustion, and impaired activation capacity [44]. In this study, SGCE was negatively correlated with activated memory CD4+ T cells, while ATP8B2 and SGCE were positively correlated with M2 macrophages. We speculate that abnormal mitophagy may mediate the imbalance of immune cell functions through these biomarkers and construct an immunosuppressive microenvironment in CRC. Tumor immunotherapy represented by immune checkpoint inhibitors has become an effective clinical strategy for the treatment of malignant tumors [45]. Inhibition of mitophagy in CRC cells impairs mitochondrial function and induces massive ROS accumulation, which upregulates PD‐L2 expression via the STAT3 pathway [46, 47]. The binding of PD‐L2 to PD‐1 inhibits mitophagy in CD4+ activated memory T cells, preventing them from maintaining metabolic reprogramming and ultimately leading to functional exhaustion [48]. Furthermore, in CRC, TGF‐β secreted by M2 macrophages suppresses CD28 expression, resulting in imbalanced mitophagy and functional exhaustion of T cells [49, 50]. Correlation analysis in this study showed that ATP8B2 was positively correlated with PDCD1LG2 and CD28. We hypothesize that mitophagy may indirectly affect the expression levels of immune checkpoints such as PDCD1LG2 and CD28 by regulating the expression of the biomarker ATP8B2, thereby further strengthening the immunosuppressive phenotype of CRC.

Single‐cell sequencing analysis in this study revealed that the expression levels of SGCE and RANGAP1 were significantly elevated in fibroblasts from CRC tissues compared with normal controls, which is consistent with the activation characteristics of CAFs in CRC. SGCE, a key molecule regulating ECM accumulation and remodeling, has been confirmed to regulate the migration and transformation of CAFs in breast cancer [51]. RANGAP1 plays a critical role in nucleocytoplasmic transport, spindle assembly, and cell cycle regulation [52], and its high expression may enhance the protumor activity of CAFs by modulating RNA transport and gene expression. We propose that abnormal mitophagy may promote excessive ECM deposition and remodeling by regulating SGCE expression in fibroblasts, driving the transformation of fibroblasts into CAFs and thereby accelerating CRC development via the secretion of protumor factors. In addition, high expression of RANGAP1 in CAFs may act synergistically with SGCE to further enhance the oncogenic properties of CAFs. However, the direct regulatory effects of mitophagy on SGCE, ATP8B2, and RANGAP1, as well as the synergistic mechanism of these three genes in CAFs activation, still require further functional experimental verification.

Furthermore, the odds ratios (ORs) of the biomarkers (SGCE, ATP8B2, and RANGAP1) in MR analysis were all close to 1. Although statistically significant, their effect sizes were relatively small. This phenomenon is common in genetic studies of complex diseases such as CRC: the occurrence and progression of complex diseases are usually driven by the synergistic effects of multiple genes and multiple pathways. The independent contribution of a single gene to disease risk is often limited, and such genes are more likely to act as risk modifiers or network regulatory nodes rather than potent single drivers [53, 54]. Combined with the results of the GGI network analysis in this study, the three biomarkers showed potential interactions with 66 genes (e.g., SGCE with PPP1R9A, RANGAP1 with PEG10), suggesting that they may cooperatively affect CRC progression by participating in a polygenic regulatory network. Meanwhile, scRNA‐seq results revealed that they were differentially expressed in specific cell types of the CRC TME (e.g., fibroblasts, B cells, T cells) and were closely associated with immune cell infiltration and immune checkpoints, further supporting their functional roles in the immune regulatory network of CRC.

In conclusion, SGCE, ATP8B2, and RANGAP1 could serve as potential mitophagy‐related biomarkers with genetically supported causal relevance to CRC development, providing a theoretical basis for the screening of CRC diagnostic biomarkers and therapeutic targets. However, this study still has some limitations. First, MR infers causal relationships using SNPs, which reflects the potential impact of genetically regulated biomarker expression on CRC risk, rather than validating the direct biological functions of genes during CRC progression. Future functional experiments are therefore required to elucidate the molecular mechanisms underlying these genetic associations. Second, the relatively small size of the clinical sample may compromise the generalizability of our findings. Subsequent studies will expand the clinical sample size to further validate the clinical expression characteristics of these biomarkers. Third, owing to the significant tissue specificity of eQTLs, eQTL data derived from peripheral blood may not fully reflect the genetic regulatory patterns of gene expression in colonic tissue, which could introduce potential bias into causal inference. Future studies should employ colon tissue‐specific eQTL datasets to validate the causal associations, thereby enhancing the tissue relevance and reliability of the research findings.

## 4. Materials and Methods

### 4.1. Data Collection

The Cancer Genome Atlas (TCGA)‐colorectal adenocarcinoma (COAD) cohort retrieved from TCGA database (https://tcga-data.nci.nih.gov/tcga/) was utilized as a training dataset. This cohort comprised tissues from 471 CRC patients and 41 normal controls. The GSE9348 (GPL570) as a testing dataset included tissues from 70 CRC patients and 12 normal controls and was sourced from the Gene Expression Omnibus (GEO) database (https://www.ncbi.nlm.nih.gov/geo/). Meanwhile, a scRNA‐seq profile GSE200997 (GPL21697) was also collected in the GEO database, including the tissues from 16 CRC patients and seven normal controls. Additionally, three mitophagy‐related pathways (R‐HAS‐5205647, R‐HAS‐5205685, and R‐HAS‐8934903), including 28 MP‐RGs (MP‐RGs), were collected from the Reactome (https://reactome.org/) database. Genome‐wide association study (GWAS) data (ukb‐b‐20145) of CRC and the expression quantitative trait loci (eQTL) data of the mitophagy‐related differently expressed genes (DE‐MPGs) were got from the Integrative Epidemiology Unit (IEU) Open GWAS database (https://gwas.mrcieu.ac.uk/). The UKB‐b‐20145 contained 9,851,867 SNPs from the 462,933 (ncase: ncontrol = 1494: 461,439) European samples. All the data were downloaded on November 20, 2023.

### 4.2. Identification of DEGs

It started with screening the DEGs in TCGA‐COAD between CRC and controls using the DESeq2 (v. 1.38.0) package (|log2Fold Change (FC)| > 1, adj *p* < 0.05) [55]. Particularly, the ggplot2 (v. 3.4.1) and ComplexHeatmap (v. 12.14.0) packages were separately applied to draw the volcano plot and heatmap for displaying DEGs [56, 57].

### 4.3. Weighted Gene Coexpression Network Analysis (WGCNA)

According to the 28 MP‐RGs, gene set variance analysis (GSVA) scores of samples in TCGA‐COAD was computed via the ssGSEA algorithm from the GSVA (v.1.46.0) package. Afterward, the Wilcoxon test contrasted the GSVA score differences between CRC and controls (*p* < 0.05). Next, a coexpression network was developed by the WGCNA (v. 1.71) package [58]. Before this, the outliers in all samples were filtered in TCGA‐COAD. The identification of soft threshold (*β*) was based on the *R* ^2^ (*R* ^2^ > 0.85) and mean connectivity (close to 0). Based on the dynamic tree cutting, all genes were divided into several distinct gene modules, each containing at least 100 genes. The Spearman correlation coefficients (cor) of each module with the GSVA scores were calculated. Among them, the modules with the highest positive and negative correlation were named as key modules (|cor| > 0.3, *p* < 0.05). Key module genes were determined based on the |module membership (MM)| > 0.8 and |gene significance (GS)| > 0.2. Ultimately, DE‐MPGs were achieved by intersecting DEGs and key module genes.

### 4.4. Two‐Sample MR Analysis

For identifying mitophagy‐related candidate genes with potential causal relevance to CRC, two‐sample MR analysis was performed utilizing the TwoSampleMR (v. 0.5.6) package [59]. DE‐MPGs were converted to Ensembl IDs and mapped to the corresponding eQTL exposure identifiers in the OpenGWAS database. The eQTL datasets in question were sourced from the eQTLGen Consortium and generated from a meta‐analysis of gene expression eQTLs using peripheral blood samples from approximately 31,684 individuals of European ancestry. CRC (ukb‐b‐20145) was an outcome factor. Genetic variant SNPs were used as IVs. IVs were strongly associated with exposure factors but not with confounders. An equally important criterion was that IVs must be directly affected only by outcome factors, not other factors. Based on the above criteria, the IVs were screened by extract_instruments function (*p* < 5 × 10^−6^, clump = TRUE; *r*2 = 0.001, kb = 100) and the extract_outcome_data function (proxies = TRUE, rsq = 0.8). The F‐statistic of each IV was computed as F‐statistic = *β* ^2^/SE^2^, where *β* represents the effect size of the SNP on the gut microbiota, while SE denotes the standard error (SE) of *β*. Weak IVs with *F* < 10 and SNPs < 3 were excluded. The MR‐PRESSO method was employed for outlier SNP detection, and SNPs with *p* < 0.05 were filtered out. After screening the IVs, the causal correlations between the DE‐MPGs and CRC were explored based on five algorithms (NbDistribution = 1000) (MR Egger test [60], weighted median [61], inverse variance weighted (IVW) test [62], simple mode [59], and weighted mode [63]), and the IVW test served as a main method (*p* < 0.05). The weight for each SNP in IVW was determined by its precision: $w_{i}=1/{\text{SE}}_{i}^{2}$, where *w*_*i*_ represents the weight of the *i*th SNP, and SE_i_ denotes the SE of the *i*th SNP’s effect estimate outcome. In particular, scatter, forest, and funnel plots were used to show these results. Specifically, based on the genetic effects of SNPs, the mr_scatter_plot function was employed to visualize the associations between DE‐MPGs and CRC through scatter plots. Additionally, the mr_forest_plot function was used to generate forest plots, evaluating the diagnostic utility of SNP loci within the DE‐MPGs for CRC. Furthermore, the mr_funnel_plot function was used to perform a random‐effects analysis to assess the randomness of the results, generating funnel plots.

Genes with OR > 1 were risk factors for CRC, while genes with OR < 1 were defined as protective factors.

### 4.5. Sensitivity Analysis

Here, we further verified the MR results via the TwoSampleMR (v. 0.5.6) package [63]. Cochran’s *Q* test was applied to check for heterogeneity by mr_heterogeneity function, and *p* > 0.05 (IVW and MR Egger tests) represented that there was no heterogeneity in the results. Using mr_pleiotropy_test function, the horizontal pleiotropy was carried out, and *p* > 0.05 represented that there was no pleiotropy in the results. Leave‐one‐out (LOO) analysis was used to examine effect of each SNP on outcome. Moreover, the MR Steiger test again determined the causal correlations between the IVs and CRC (*p* < 0.05). Ultimately, genes that passed the above tests were considered candidate genes for subsequent analysis.

### 4.6. Function Enrichment Analyses and GGI Network

We further explored the potential roles of mitophagy‐related candidate genes in CRC. Specifically, Gene Ontology (GO) analysis was performed using clusterProfiler (v. 4.7.1.3) [64] and org.Hs.eg.db (v. 3.18.0) (https://bioconductor.org/packages/org.Hs.eg. db/) packages (adj *p* < 0.05). In addition, genes that interacted with candidate genes were investigated by the GeneMANIA (https://www.genemania.org/) database, and a GGI network was built.

### 4.7. Machine Learning

The mitophagy‐related candidate genes underwent further screening. The feature genes were severally determined by LASSO regression and support vector machine‐recursive feature elimination (SVM‐RFE) analyses using glmnet (v 4.1.4) [65] and caret (v. 6.0.93) (https://CRAN.R-project.org/package=caret) packages. Later, the hub genes were obtained by overlapping the feature genes of the above machine learning. The expression levels of CRC and controls were compared in training and testing datasets by the Wilcoxon test (*p* < 0.05). Furthermore, receiver operating characteristic (ROC) curves of hub genes were generated to judge the diagnostic abilities via the pROC (v. 1.18.0) package (area under curves (AUCs) > 0.7) [66]. Eventually, the screening of mitophagy‐related biomarkers should meet the following conditions: (1) genes exhibiting significantly different expression in CRC within both training and testing datasets,(2) genes demonstrating consistent expression trends within both training and testing datasets and (3) genes with AUCs exceeding 0.7 in both training and testing datasets.

### 4.8. Nomogram Establishment

In TCGA‐COAD, a nomogram integrating mitophagy‐related biomarkers was established via rms (v. 6.5.0) package (https://CRAN.R-project.org/package=rms). More specifically, total points of biomarkers were used to predict the CRC risk. ROC curve and decision curve analysis (DCA) were applied to assess the diagnostic performance of this nomogram model.

### 4.9. GSEA

For investigating crucial functional pathways of mitophagy‐related biomarkers, GSEA was applied. Here, “c2.cp.kegg.v7.2.symbols.gmt” obtained from the molecular signatures database (MSigDB; https://www.gsea-msigdb.org/gsea/msigdb) served as a background gene set. Spearman’s cor between biomarkers and other genes were utilized to sort genes in descending order. Subsequently, GSEA was performed on each biomarker through clusterProfiler (v. 4.7.1.3) [64] and org.Hs.eg.db (v. 3.18.0) packages (adj.*p* < 0.05).

### 4.10. Evaluation of Infiltrating Immune Cells and Immune Checkpoints

Through CIBERSORT, infiltration profiles of 22 immune cells in TCGA‐COAD was analyzed (samples with *p* > 0.05 were excluded). Next, the differential immune cells with significant differences in infiltration between CRC and controls were obtained by the Wilcoxon test (*p* < 0.05). Spearman’s correlation analysis was carried out between mitophagy‐related biomarkers and differential immune cells (|cor| > 0.3, *p* < 0.05). Additionally, 73 immune checkpoints were gathered from the published literature [67]. The expression levels of immune checkpoints were contrasted between CRC and controls by the Wilcoxon test (*p* < 0.05). Likewise, Spearman’s correlation analysis was carried out between biomarkers and immune checkpoints with significant differences (|cor| > 0.3, *p* < 0.05).

### 4.11. Potential Drug Prediction

To screen the potential drugs for mitophagy‐related biomarkers, we predicted the drugs on the Description: Drug Signatures Database (DSigDB, https://tanlab.ucdenver.edu/DSigDB) using each biomarker as a keyword. Furthermore, a drug–gene network was built based on the potential drugs and biomarkers.

### 4.12. Single‐Cell Transcriptome Analysis

At the single‐cell level, mitophagy‐related biomarker expression levels in distinct cells were further explored. Initially, Seurat (v. 4.3.0) package was applied to filter GSE200997 data [68]. First, cells with detected genes < 200 genes and genes covered by cells < 3 were excluded. Whereafter, high‐quality cells and genes with more stringent standards were selected based on the more stringent standards (200 ≤ nFeature_RNA ≤ 2500; percent.mt < 5%). After normalizing the data, the top 2000 highly variable genes (HVGs) were determined via the FindVariableFeatures function. Then, the principal component analysis (PCA) was applied to observe if there were significant outlier samples, and then, the applicable principal components (PCs) were screened. Based on this, uniform manifold approximation and projection (UMAP) clustering was conducted via FindNeighbors and FindClusters functions (resolution = 0.4), and then, several cell clusters were formed. Marker genes of cell clusters were selected based on the FindAllMarkers function (only.pos = TRUE, min.pct = 0.25). Importantly, the cell subgroups were annotated based on the published literature [69] and the CellMarker2.0 website (https://bio-bigdata.hrbmu.edu.cn/CellMarker/). The differential proportion of each cell subgroup between CRC and controls was analyzed by the Wilcoxon test (*p* < 0.05). The expression levels of biomarkers in cell subgroups were analyzed.

### 4.13. Clinic Samples Collection, RNA Extraction, and qPCR

To verify the expression of mitophagy‐related biomarkers in clinical samples, qPCR analyses of biomarkers were performed. Five tumors and five paracarcinoma tissue of CRC patients were collected from Shandong Provincial Hospital Affiliated to Shandong First Medical University. This study was approved by the Ethics Committee of the hospital. Specifically, the total RNA was extracted by the TRIzol reagent (Ambion, Shanghai, China). Subsequently, reverse transcription was completed by the First Strand cDNA Synthesis‐kit (Servicebio, Wuhan, China). The primers of mitophagy‐related biomarkers and GAPDH (reference gene) are listed in Supporting Table S7. Remarkably, qPCR assay was performed on a CFX96TM Real‐Time PCR Detection System (BIO‐RAD, USA). Finally, the 2^−ΔΔCT^ method was used for relative expression comparison [70].

### 4.14. Statistical Analysis

Bioinformatics analyses were conducted by R (v. 4.2.3) software. Network was drawn using the Cytoscape (v.3.7.2) software (https://cytoscape.org/). Statistical significance across groups was confirmed by *p* < 0.05 (two‐tailed).

**Nomenclature**
CRC: Colorectal cancer
MP‐RGs: Mitophagy‐related genes
WGCNA: Weighted gene coexpression network analysis
DE‐MPGs: Mitophagy‐related differentially expressed genes
MR: Mendelian randomization
ROC: Receiver operating characteristic
scRNA‐seq: Single‐cell RNA sequencing
qPCR: quantitative PCR
SNPs: Single‐nucleotide polymorphisms
IVs: Instrumental variables
RCTs: Randomized controlled trials
GWAS: Genome‐wide association study
TCGA: The Cancer Genome Atlas
COAD: Colorectal adenocarcinoma
GEO: Gene Expression Omnibus
eQTL: Expression quantitative trait loci
IEU: Integrative Epidemiology Unit
GSVA: Gene set variance analysis
MM: Module membership
GS: Gene significance
IVW: Inverse variance weighted
OR: Odds ratio
LOO: Leave‐one‐out
GGI: Gene–gene interaction
GO: Gene Ontology
LASSO: Least Absolute Shrinkage and Selection Operator
SVM‐RFE: Support vector machine‐recursive feature elimination
AUCs: Area under curves
DCA: Decision curve analysis
MSigDB: Molecular signatures database
DSigDB: Drug Signatures Database
HVGs: Highly variable genes
PCA: Principal component analysis
PCs: Principal components
UMAP: Uniform manifold approximation and projection
CMP: Common myeloid progenitor
CAFs: Cancer‐associated fibroblasts
TME: Tumor microenvironment

## Author Contributions

Jingyi Zhao and Sining Wang contributed to data curation and analysis. Meng Kong and Jingyi Zhao contributed to the clinic samples collection and quantitative PCR. Jingyi Zhao, Zhixin Cao, and Xiangguo Tian contributed to writing the paper.

## Funding

This work was supported by the National Natural Science Foundation of China under Grant No. 82201293 and the Youth Science Foundation Cultivation Support Program of Shandong First Medical University under Grant No. 202201‐061.

## Ethics Statement

The study was conducted according to the guidelines of the Declaration of Helsinki and approved by Shandong Provincial Hospital Affiliated to Shandong First Medical University.

## Conflicts of Interest

The authors declare no conflicts of interest.

## Supporting Information

Additional supporting information can be found online in the Supporting Information section.

## Supporting information


**Supporting Information 1** Supporting Figure S1: Sample of TCGA‐COAD clustering and trait heatmap.

**Supporting Information 2** Supporting Figure S2: Forest map of Mendelian randomization (MR) analysis of seven exposure factors (genes).

**Supporting Information 3** Supporting Figure S3: Funnel plot of random judgment analysis of seven exposure factors (genes).

**Supporting Information 4** Supporting Figure S4: Leave‐one‐out forest plot of seven exposure factors (genes).

**Supporting Information 5** Supporting Figure S5: Hub gene expression levels in training and testing datasets. (a) Diagram of hub gene expression levels in training datasets. (b) Diagram of hub gene expression levels in testing datasets.

**Supporting Information 6** Supporting Figure S6: ROC diagram of the hub gene in the training dataset.

**Supporting Information 7** Supporting Figure S7: ROC diagram of the hub gene in the testing dataset.

**Supporting Information 8** Supporting Figure S8: Bubble plot of marker gene expression across different cell clusters.

**Supporting Information 9** Supporting Figure S9: Violin graph of biomarkers expressed in a single cell.

**Supporting Information 10** Supporting Figure S10: The UMAP map of biomarker expression in cell types.

**Supporting Information 11** Supporting Table S1: The result of Mendelian randomization (MR) analysis.

**Supporting Information 12** Supporting Table S2: The result of MR Steiger filtering.

**Supporting Information 13** Supporting Table S3: The result of heterogeneity analysis of seven exposure factors (genes).

**Supporting Information 14** Supporting Table S4: The result of horizontal pleiotropy analysis of seven exposure factors (genes).

**Supporting Information 15** Supporting Table S5: The result of GO analysis of seven candidate genes.

**Supporting Information 16** Supporting Table S6: The result of GGI network analysis of seven candidate genes.

**Supporting Information 17** Supporting Table S7: The result of the primers of mitophagy‐related biomarkers and GAPDH (reference gene).

## Data Availability

The data generated during the current study are available in the TCGA database (https://tcga-data.nci.nih.gov/tcga/). Gene Expression Omnibus (GEO) database (GSE9348; GSE200997) (https://www.ncbi.nlm.nih.gov/geo/), the Reactome (https://reactome.org/) database, Integrative Epidemiology Unit (IEU) Open GWAS database (https://gwas.mrcieu.ac.uk/), org.Hs.eg.db (v.3.18.0) (https://bioconductor.org/packages/org.Hs.eg.db/) packages, the GeneMANIA (https://www.genemania.org/), caret (v. 6.0.93) (https://CRAN.R-project.org/package=caret) packages, rms (v. 6.5.0) package (https://CRAN.R-project.org/package=rms), the molecular signatures database (MSigDB; https://www.gsea-msigdb.org/gsea/msigdb), the Description: Drug Signatures Database (DSigDB, https://tanlab.ucdenver.edu/DSigDB), the CellMarker2.0 website (https://bio-bigdata.hrbmu.edu.cn/CellMarker/), and the Cytoscape (v.3.7.2) software (https://cytoscape.org/).
