# Supplementary figures and images for: Identification and Validation of Mitophagy‐Related Biomarkers in Colorectal Cancer: An Integrated Analysis of Single‐Cell Transcriptome and Mendelian Randomization
Source: Genet Res (Camb). 2026 May 11;2026:5579542. doi: 10.1155/genr/5579542 (PMC13158581; doi:10.1155/genr/5579542)

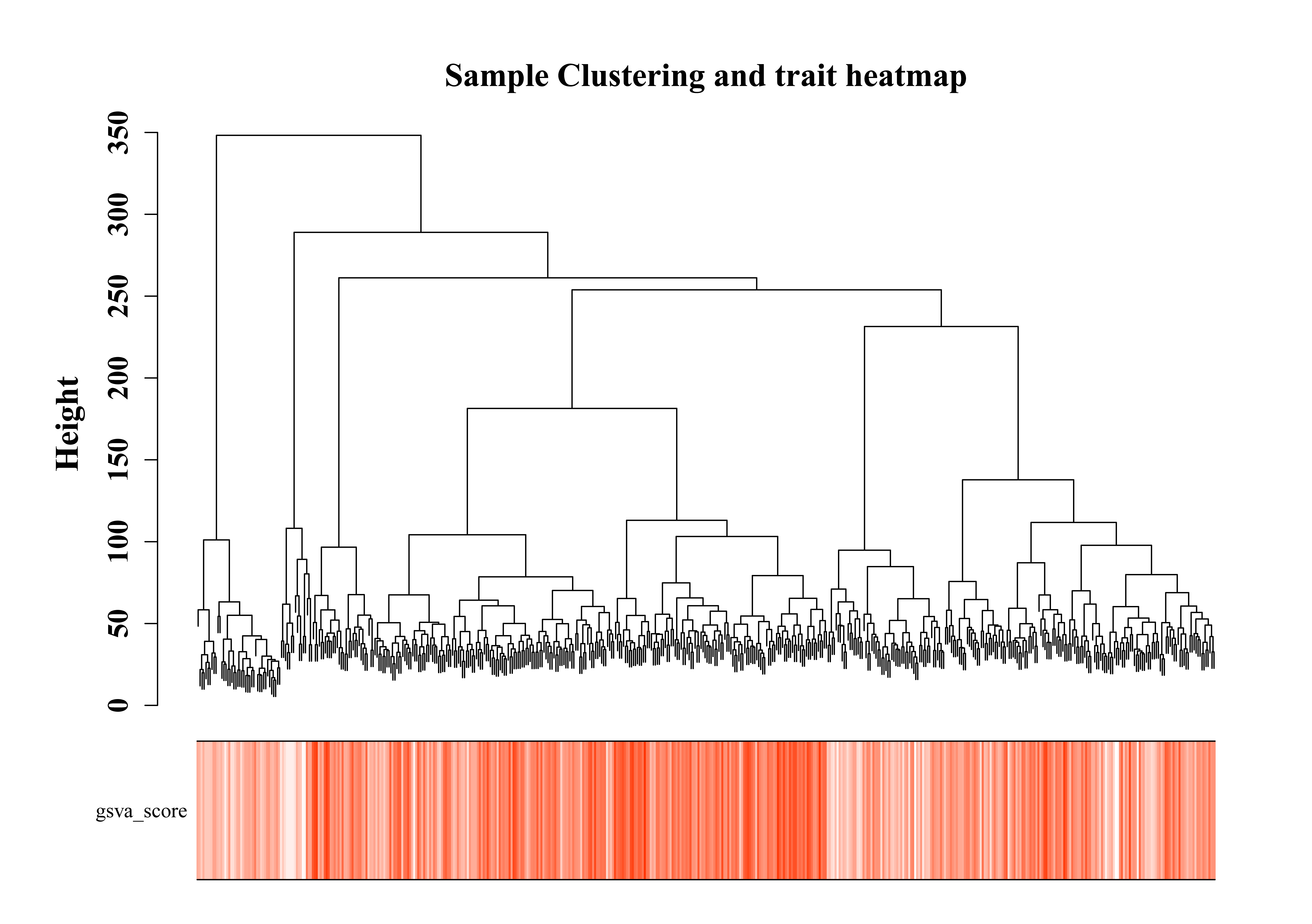

Supplement: Supplementary file 1 — Supporting Information 1 Supporting Figure S1: Sample of TCGA‐COAD clustering and trait heatmap. [file GENR-2026-5579542-s016.tif]

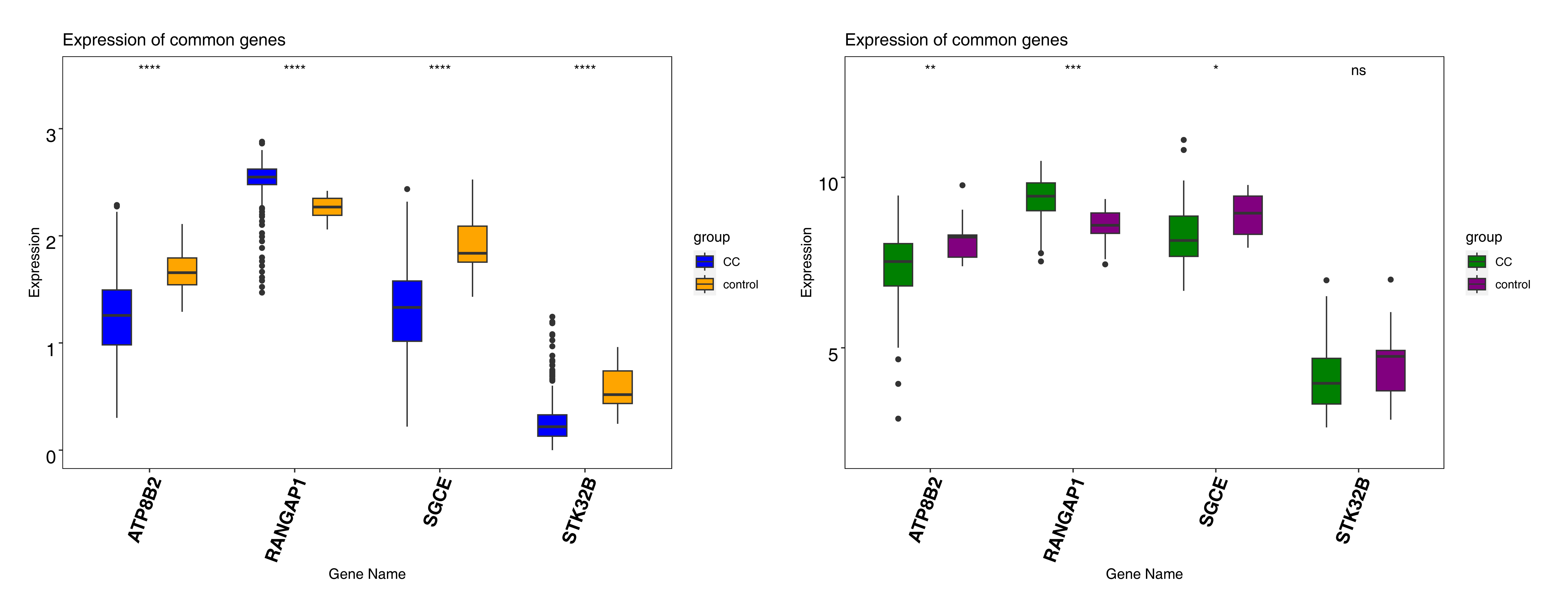

Supplement: Supplementary file 5 — Supporting Information 5 Supporting Figure S5: Hub gene expression levels in training and testing datasets. (a) Diagram of hub gene expression levels in training datasets. (b) Diagram of hub gene expression levels in testing datasets. [file GENR-2026-5579542-s013.tif]

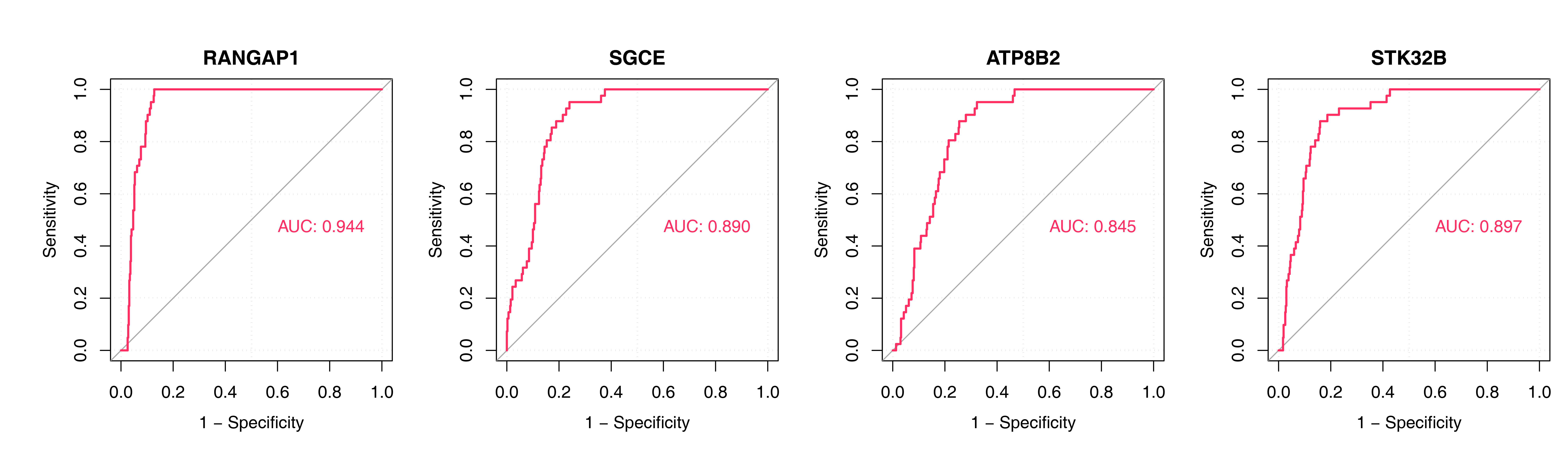

Supplement: Supplementary file 6 — Supporting Information 6 Supporting Figure S6: ROC diagram of the hub gene in the training dataset. [file GENR-2026-5579542-s012.tif]

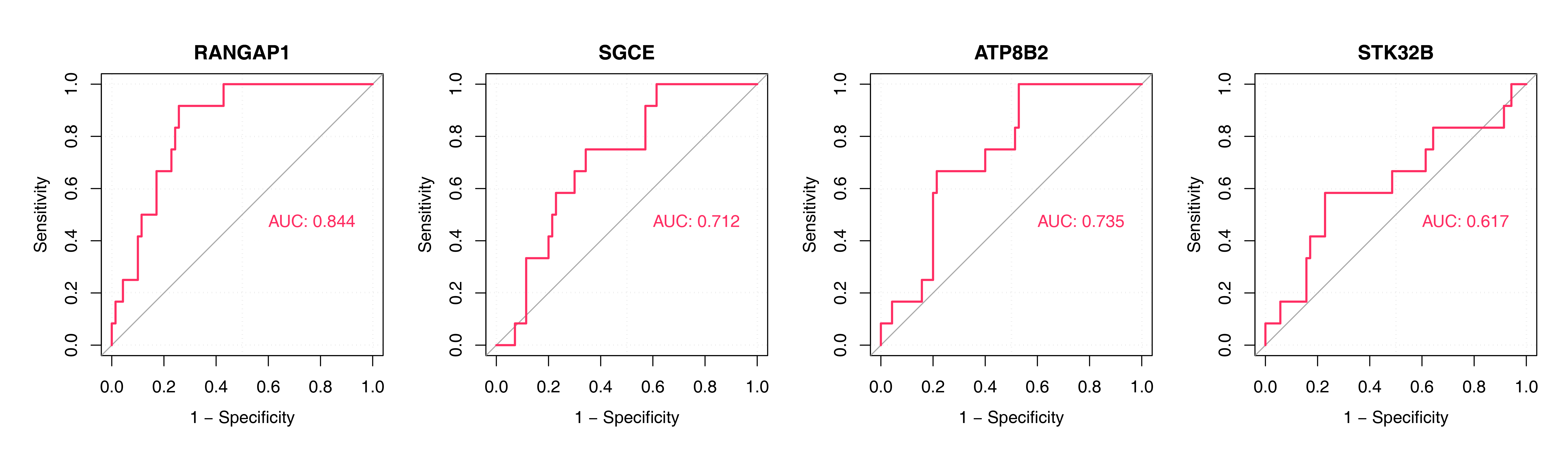

Supplement: Supplementary file 7 — Supporting Information 7 Supporting Figure S7: ROC diagram of the hub gene in the testing dataset. [file GENR-2026-5579542-s011.tif]

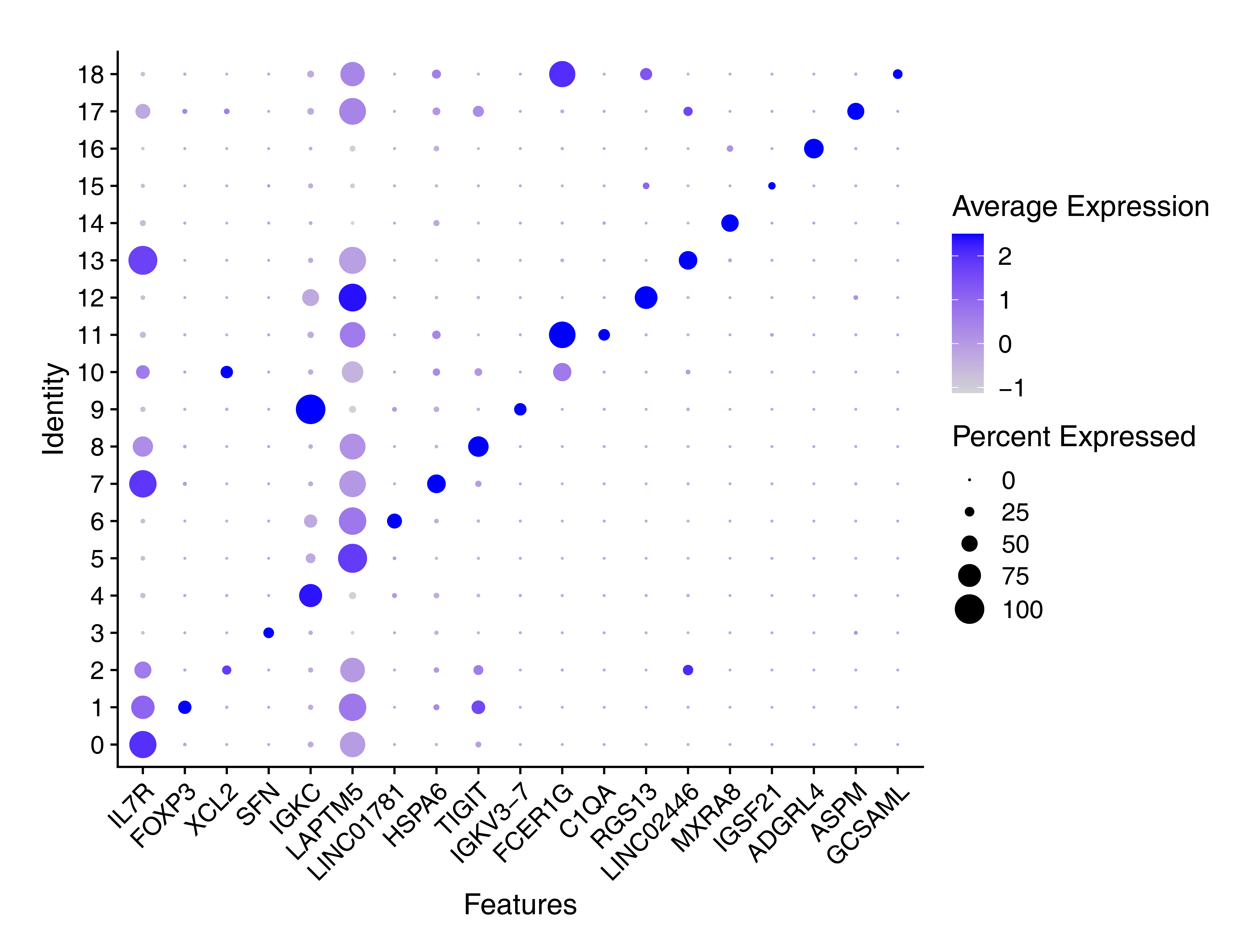

Supplement: Supplementary file 8 — Supporting Information 8 Supporting Figure S8: Bubble plot of marker gene expression across different cell clusters. [file GENR-2026-5579542-s009.tif]
